# Coach and teacher alignment in the context of educational change

**DOI:** 10.1007/s10833-025-09528-1

**Published:** 2025-05-26

**Authors:** Ethan P. Smith, Laura M. Desimone

**Affiliations:** 1 College of Education, Sport, and Human Sciences, Washington State University Tri-Cities, 2710 Crimson Way, Richland, WA 99354 USA; 2 College of Education and Human Development, School of Education, University of Delaware, Newark, USA

**Keywords:** Mathematics teacher education, Instructional coaching, Professional development, Curriculum, Education reform

## Abstract

Professional learning (PL) programs increasingly rely on the expertise of instructional coaches in supporting teacher learning and instructional change. To continue to improve the use of coaching as a lever of teacher PL, it is important to understand how both coaches and teachers understand and view their interactions in the context of such PL programs. Using survey data, we investigated middle school mathematics teacher and coach perceptions about the quality of their PL and the curriculum materials embedded within that PL, and considered individualized coaching approaches that may have influenced teachers’ perceptions. We found that coaches had more positive views about the quality of district-mandated professional learning and curriculum materials compared to teachers. However, we also found that teachers who had coaching support viewed their learning experiences and curriculum materials more positively than teachers who did not have a coach. Finally, we found that teachers’ perceptions about the quality of their coach related to their perceptions about the quality of their PL and curriculum, and that coaches’ reported emphasis on using curriculum materials in their coaching work was related to more negative teacher perceptions about the quality of that curriculum and associated PL. These results indicate meaningful differences in how teachers and coaches perceive of their shared work, suggest the importance of a coach’s perceived expertise and interpersonal relationship with their teachers, and point to challenges and complexities in how teachers and coaches can mutually adapt their shared work to achieve desired instructional change.

## Introduction

Although advances in the field suggest core design principles for teachers’ professional learning (Desimone, 2009; Kennedy, 2016), the structure of professional learning (PL) programs continues to vary across the educational landscape. One key component that is increasingly drawn on by PL programs is content-focused instructional coaching (Darling-Hammond, 2017; Ippolito et al., 2021; Korthagen, 2017; Kraft et al., 2018). However, the inclusion of coaching in PL models has also shown mixed results in improving fidelity and integration with PL structures and goals—especially in the context of mathematics (Campbell & Griffin, 2017; Garet et al., 2010; Harbour & Saclarides, 2020; Kraft & Blazar, 2017; Lynch et al., 2019; Mills et al., 2020). Hjalmarson and Baker (2020) discuss how this may be in part because such coaches are “hidden players” (p. 52) in studies of PL—their work falls largely outside of the controlled PL structures and their own knowledge and views relevant to the PL aims are rarely captured to the same extent as those of teachers. These missing perspectives matter in part because the scalability of coaching models rests not merely on the PL design itself, but also on the ability of coaches and teachers to mutually adapt their shared work to support their particular context and needs (Russell et al., 2020). Coaching entails a more informal negotiation between coaches and teachers about how to enact more formal instructional improvement goals within the classroom context (Kochmanski & Cobb, 2023), and such informal interactions may be key in not only changing teachers’ instructional beliefs but also their *practices* in support of educational reform (Shirrell et al., 2019). Because coaching tends to be employed along with complementary interventions such as district PL workshops and reform-oriented curriculum materials (Kraft et al., 2018), and because local interactions between teachers and coaches inform their understanding of and adaptation of such educational infrastructure (Hopkins & Woulfin, 2015; Kochmanski & Cobb, 2023; Russell et al., 2020), it is critical to understand the perceptions of teachers and coaches with regard to these complementary interventions and with regard to one another.

The purpose of this paper is to center teacher and coach perspectives about the quality and attributes of district-mandated PL and associated curriculum materials, and to investigate how teacher- and coach-reported aspects of the coaching work influences their perceptions about these aspects. We contend that, to understand how coaching impacts teachers’ experience with their PL, it is vital to uncover and compare the perspectives of both the teacher *and* their coach, and to recognize the individualistic nature of how coaches may be supporting their teachers’ learning regardless of the intended approach of an associated PL program.

## Literature review

### Mathematics coaching to support teacher learning

Instructional coaching from content-area specialists is an increasingly common delivery method of PL programs for mathematics teachers (Lynch et al., 2019). Coaching is especially appealing within such programs because it has the potential to attend to features of high-quality PL, including content focus, active learning, sustained duration, coherence, and collective participation (Desimone & Pak., 2017). However, less is known about how coaching actually contributes to goals of PL programs (Hjalmarson & Baker, 2020), or if a certain approach toward coach training or coaching focus might best influence teacher practice (Blazar & Kraft, 2015).

One issue is that the definition of a term such as “mathematics coaching” can vary considerably across educational contexts, from describing school-based coaches focused on teacher observation and feedback to district-based specialists focused on providing PL around ambitious instruction (Cobb & Jackson, 2011). Activities offered by coaches can also vary not only across but within schools and districts, as coaches are often responsive to the specific needs of the teachers whom they support (Campbell & Griffin, 2017; Russell et al., 2020). Although the highly responsive nature of coaching may make it harder to study coaching in the context of PL (Hjalmarson & Baker, 2020), it may also be a key aspect of what makes coaching effective. Indeed, the extent to which coaching activities adhere to teachers’ perceived individual needs has considerable influence on whether such activities achieve their goals (Desimone et al., 2014; Feiman-Nemser, 2001; Polikoff et al., 2015). It is thus critical for programs aimed at instructional change to be perceived as legitimate, relevant, and actionable across all organizational levels, including both coaches and teachers (Adolfsson, 2024). Additionally, mathematics coaches working in schools full-time have been found to be more likely to foster instructional change compared to coaches who are part-time or work at the district level (Coburn & Russell, 2008; Harbour & Saclarides, 2020), further suggesting the importance of interpersonal trust and responsiveness with coaches and their teachers and school leaders. In essence, coaches are not merely a passive conduit through which a school district or PL partner fosters instructional change, but rather are integral members of the school community who help to negotiate (rather than dictate) a shared vision of mathematics instruction with the teachers whom they support.

The individualized and responsive nature of coaching warrants careful examination of how school or district goals around PL translate from the coach perspectives of their role, to the coach’s work with teachers, to changes in those teachers’ instructional practices. Coburn and Russell (2008) provide an insightful account of how different approaches to coach training can in turn impact the coaching work and teacher experience with their coaching. They examined efforts by two school districts that were aiming to support the roll-out of new ambitious mathematics curricula and described how the approach toward the coaching role resulted in starkly different interactions between teachers, their peers, and their coaches. Because PL activities for coaches in one school district focused on direct explanation and use of curriculum materials, those coaches’ interactions with teachers—and, in turn, teachers’ interactions with one another—tended to be “low” depth (i.e., emphasizing lesson planning, use of curriculum materials, pacing, etc.). In the other district, coach PL focused on task analysis and analyzing student strategies, resulting in coaches emphasizing—and their teachers more commonly displaying—high depth interactions which “addressed underlying pedagogical principles of the approach, the nature of the mathematics, and how students learn” (Coburn & Russell, 2008, p. 212). These authors note how “coaches acted as bridges between districts and schools, bringing routines of interaction into teachers’ social networks, which then diffused amongst teachers” (Coburn & Russell, 2008, p. 219). Such findings are bolstered by Sun et al. (2014), who found that coach expertise with mathematical knowledge for teaching may help to “augment the extent to which teachers can learn from interacting with close colleagues” (p. 21). In such ways, coaches supported with a clear understanding of and approach toward fostering high-depth interactions (around curriculum materials, for instance) may be better positioned in fostering similar high-depth interactions with and among the teachers whom they support.

Bengo (2016) adds to such findings by indicating that effective coaching (i.e., coaching that convinces teachers to adapt new instructional practices) depends upon both teachers’ interpersonal trust in their coach (e.g., affective qualities of the coaching) and teachers’ belief that their coach holds content and pedagogical expertise. Blazar (2020) similarly suggests that coaching quality rests upon not only the coach’s content and teaching expertise, but also “coaches’ relationships and rapport with teachers and other school and district staff” (pp. 6–7). Yopp and colleagues (2019) also found that coaches’ self-assessment of their coaching skills, rather than their assessed mathematical knowledge for teaching, was positively related to their teachers’ increase in mathematical knowledge for teaching, self-efficacy, and changes in instructional practices. Such diverse findings suggest that building both a robust and aligned vision of mathematics instruction between teachers and coaches—what Coburn and Russell (2008) refer to as “Congruence” (p. 229)—appears to be an important aspect of the coaching work. Effective coaching rests not merely on coach expertise, but also the *perceptions* of that expertise (from both teachers and coaches) and the strength of interpersonal relationships that teachers have with their coaches.

### Divergence of teacher-coach perceptions

It is, of course, not guaranteed that teachers will align with their coaches regarding a vision of mathematics instruction and goals of instructional change. Indeed, evidence suggests that mathematics coaches’ vision of and approach towards mathematics instruction is often distinct from that of teachers. Campbell and Malkus (2014), for instance, describe how coaches in their study developed more positive perceptions of ambitious mathematics instruction (and an aligned PL program) as they transitioned from previous roles as teachers. After these coaches participated in carefully designed, conceptual- and inquiry-focused coaching PL, they effectively became greater “cheerleaders” for this sort of PL approach compared to when they were teachers. Such results are echoed by Swars et al. (2018), who describe the challenges of shifting the beliefs, content knowledge, and teaching practices of elementary teachers as they engaged with a mathematics specialist endorsement program. These studies emphasize how teachers can benefit from explicit professional learning in developing relevant coaching skills and mindsets. However, these findings also suggest that teachers—even those interested in becoming mathematics coaches—often hold less precise understandings aligned with efforts of instructional reform.

While a divergence between teacher and coach perceptions about their shared work may appear beneficial if the goal is to *spur* such instructional change, it can run counter to the collaborative and responsive aspects of effective coaching previously described. Indeed, if teachers are not bought in to a coaching activity, this could present an additional challenge—in addition to effectively executing the activity itself—for the coach (e.g., Kane & Saclarides, 2022). The ability of teachers and coaches to mutually adapt their practices effectively—to find common ground in their approaches while maintaining the aims of the associated PL program—may be key in the scalability of such programs (Russell et al., 2020). Persistent, divergent perspectives between teachers and coaches about district policy (such as district-mandated PL or curriculum use) could thus present a challenge to coaches tasked with supporting such policies.

Taken together, this evidence shows that coaching as a feature of teacher PL is difficult to accurately describe and effectively execute. While there are indications about the sorts of coaching activities that can support teacher learning (e.g., Gibbons & Cobb, 2017), it is not well understood whether, when, and how such activities support strengthening participant perceptions of associated PL programs and curricula. While fostering an aligned vision of instruction with teachers appears to be a key feature of effective coaching, aspects inherent to the coaching role may lead to diverging perspectives on pedagogy and curriculum between teachers and their coaches. Understanding how teachers’ and coaches’ perspectives about their districts’ PL and curriculum decisions relate to the teacher-coach work can help us begin to untangle this complexity inherent in coaching.

### Conceptual framework

We draw on the policy attributes theory (Porter, 1994) to investigate teacher and coach perceptions of curriculum-embedded PL aimed at mathematics for students aged 10–14. This theory posits that implementation quality is a function of the policy’s specificity (level of detail and clarity), consistency (alignment with other elements of the policy system), authority (resources and buy-in), power (rewards and sanctions associated with the policy), and stability (of actors and the policy itself). In our present study, the two policies under investigation are the PL program and associated mathematics curricula, which are also common complementary features employed alongside teacher coaching (Kraft et al., 2018). Additionally, this age range (10–14) was chosen in part because of the critical role that such transitional periods have in relation to student motivation and engagement in school (Madjar et al., 2018).

Importantly, we focus on how both teachers and coaches perceive the attributes rather than how the attributes are codified in the PL design, given that it is how actors interpret and understand policy that informs their behavior (Desimone, 2002). The policy attributes framework has been used to good effect in studying PL in the context of research-practice partnerships, third party-PL providers, and standards-based reform (e.g., Berends, 2000; Polikoff, 2012; Polikoff & Porter, 2014; Porter, 1986).

We hypothesize that these attributes of both the curriculum and PL, as perceived by teachers and coaches, are important indicators of the teacher-coach experience that both warrant investigation. High-quality curriculum materials and teacher PL programs are both recognized as powerful mechanisms for fostering instructional improvements (e.g., Chingos & Whitehurst, 2012). Further, decades of scholarship on school reform have established that the content of, and teachers’ experiences with, curriculum (e.g., Blazar et al., 2020; Schmidt et al., 1997) and professional learning (e.g., Fishman et al., 2003; Kennedy, 2016) are two of the most powerful levers in fostering instructional change. However, because professional learning and curriculum work in interaction to address instructional change, Hopkins and Spillane (2015) note that “focusing on any one component (e.g., curricular materials, professional learning) and its association with instructional change is inadequate” (p. 446). Additionally, it is the combination of these more formal components and informal learning opportunities (e.g., coaching) that creates ideal opportunities for teacher professional learning (Shirrell et al., 2019). Thus, an analysis of this sort of educational infrastructure must address both formal components employed within school systems as well as how such components are interpreted and adapted by school leaders and teachers (Hopkins & Woulfin, 2015). Attending to teacher and coach perceptions of *both* PL and curricula can thus better contextualize participant experiences within the school system. Given that coaches are especially well-positioned to support such school or district PL programs (Lynch et al., 2019) and/or new curricula adoption (e.g., Coburn & Russell, 2008; Obara & Sloan, 2009), understanding how the perspectives and work between teachers and their coaches relate to these policy-level aspects is also especially worthwhile.

With this in mind, our study focuses on the following research questions, in the context of middle school mathematics:

1. In what ways do mathematics teachers and coaches align or diverge in their perceptions of their professional learning and curriculum quality?
2. In what ways do mathematics teachers with and without a coach align or diverge in their perceptions of their professional learning and curriculum quality?
3. How do teacher beliefs about coach quality, and coach focus on different instructional activities, relate to the perceptions that teachers and their coaches have regarding the quality of their professional learning and curriculum?

## Methods

### Context

The data for our study were collected as part of the Research on Curricular Alignment Partnerships (R-CAP) project. This project collaborated with 12 professional learning partnerships (PLPs) situated in school districts (i.e., administrative units that provide oversight for multiple schools in the same geographical area) across the United States whose efforts to enhance equity in middle school education were funded as part of an initiative by the Bill & Melinda Gates Foundation (BMGF). Each partnership consisted of a school district and a PL provider contracting with the district. The focus of the BMGF initiative was to grow the evidence base around the effectiveness of curriculum-connected PL services, help partners and the field better understand the needs of schools serving students who are predominantly Black, Latino, dual language designated, and/or affected by poverty, and contribute to a robust PL marketplace by testing and refining curriculum-specific PL services that are effective, adoptable, and affordable. As part of the R-CAP project, we collected survey data from stakeholders within participating districts, including teachers and coaches. These survey data form the basis of the present investigation.

### Participants

Because the majority of teachers in this study were mathematics teachers, we chose to focus our investigation on this population. This approach also allowed us to explore relationships between content-specific coaching activities and teacher perceptions about the quality of their PL and curriculum. To this end, we excluded three PLPs that were focused on English/Language Arts or Science content and one PLP for which we were unable to acquire permission to survey teachers in time for this survey iteration, leaving eight participating PLPs. Given that not all teachers completed the full survey, the sample for each scale included between 415 and 432 mathematics teachers and 69 coaches. Because of the relatively low number of incomplete responses to certain scales (at most 17 out of 432 for Curriculum Power and Stability scales), and because the incomplete surveys tended to be for later items in a time-intensive survey of ~ 60 items (indicating survey fatigue), we do not believe that these incomplete responses pose a major concern. The participation rates for each partnership are shown in Table 1.

**Table 1:** Teacher and coach response rates across partnerships

Partnership	Teachers			Coaches		
	Targeted	Responses	Rate	Targeted	Responses	Rate
A	65	41	63%	5	5	100%
B	232	145	63%	20	18	90%
C	47	31	66%	23	16	70%
D	16	10	63%	2	2	100%
E	56	28	50%	2	2	100%
F	14	11	79%	4	4	100%
G	154	97	63%	18	9	50%
H	68	37	54%	15	10	67%

Because we were interested in the relationship between teacher and coach responses regarding the PL program and the work of coaches for our third research question, we also created a secondary dataset of participating teachers with matching school-based coaches. This sample gave us 191 teachers across 34 schools, with a median of 4 teachers per school. While most (29) of these schools had one participating coach, five schools had two coaches who completed the survey. In such cases we took the mean of the coach survey responses after confirming that the two coach responses did not vary by greater than 1 unit for any of the relevant scales. In this secondary “hierarchical dataset” the teacher responses represent the level 1 data and coach responses (in essence, school-level responses) formed the level 2 data.

### Measures

Our data come from both a teacher and a coach survey administered from mid-December 2020 to mid-February 2021. These surveys asked respondents a range of questions, including items concerning respondents’ perceptions of the PL program and curriculum attributes, the context of teachers’ coaching (i.e., was their coach based only at their school, serving multiple schools [district-based], or did they not have access to a coach to support their learning from the PL program), and their experiences with coaching/being coached. We recognize that these data were collected during the COVID-19 pandemic, which afforded challenges and opportunities to the work of coaches and the PLPs. This aspect is addressed further in our discussion of the study’s limitations.

Additionally, while PLPs were broadly expected to align with the foci of the initiative, the districts and PL providers were given some leeway in choosing which curriculum materials to integrate with their PL. In the United States, private developers create sets of materials to support learning standards (e.g., textbooks, teachers’ guides, student workbooks) for purchase by school districts. As such, the variety in content and quality of such materials can either support or hinder the goals of teacher learning and instructional change (Polikoff, 2015). To address such concerns, all participating PLPs were required to use curriculum materials that had been externally vetted by the nonprofit EdReports (https://edreports.org/) as meeting expectations of modern college and career readiness standards. However, because our investigation also separates policy attributes along lines of both PL and curriculum, any distinctions between participants’ perceptions of their PL and the curriculum would also be discernable in our analyses.

#### Professional learning and curriculum quality

We created multi-item scales for each policy attribute (Porter, 1994) to determine the perceived quality of the PL and the curriculum as understood by teachers and coaches. Teacher and coach perceptions of PL attributes consisted of three scales: specificity, consistency, and authority. PL stability was not included because the PL programs were newly introduced. Because these were curriculum-embedded PLPs, the power-related items focused on both the PL and curriculum and were determined by the researchers to be more relevant in describing curriculum attributes. Curriculum attributes consisted of six scales, reflecting the emphasis on understanding curriculum-embedded PL for R-CAP: specificity, consistency, institutional authority, normative authority, stability, and power. Each scale (except for curriculum stability, explained below) used the same Likert response categories (0 = Completely disagree, 5 = Completely agree). Cronbach’s coefficient alpha, which measures internal scale consistency (Wang & Osterlind, 2013, p. 36) was determined for each scale created from the survey responses to ensure reliability.

PL specificity (α = 0.923 for teacher, α = 0.843 for coach) items were adapted from Hill and Desimone (2018) and described the extent of detail and clarity within the PL, such as the extent to which the PL “has clear objectives and goals for my learning”. The curriculum specificity scales (α = 0.762 for teacher, α = 0.559 for coach) served a similar function in describing the clarity of the curriculum materials and were adapted from the School District of Philadelphia (SDP, 2021) and the Center for Standards, Alignment, and Learning (C-SAIL, 2017).

PL consistency (α = 0.749 for teacher, α = 0.582 for coach) and curriculum consistency (α = 0.893 for teacher, α = 0.819 for coach) items were adapted from SDP (2021) and focused on the alignment of policies with the PL or curriculum, such as whether the PL was “consistent with district policies (such as state standardized testing and content standards)” or whether the curriculum was “aligned to my state’s math assessments.”

PL authority (α = 0.884 for teacher, α = 0.746 for coach) items were adapted from C-SAIL (2017) and Student Achievement Partners (SAP, 2019) and described the amount of resources devoted to the PL (institutional authority, e.g. “I feel supported by my school leadership in implementing what I learned”) as well as participant buy-in (normative authority, e.g., “My participation in professional learning on my curriculum can lead to better achievement outcomes for students”). Because of the finer comb investigation on curriculum materials in R-CAP, we created curriculum scales for both institutional authority (α = 0.784 for teacher, α = 0.678 for coach) and normative authority (α = 0.741 for teacher, α = 0.783 for coach), items which were adapted from C-SAIL (2017) and SAP (2019) as well as Marsh et al. (2005). The institutional authority scale included Likert scale items such as “My district has made using the curriculum a majority priority” while the normative authority scale included items such as “The curriculum includes more content than can be covered adequately in the schools year.”

The curriculum stability scale (α = 0.838 for teacher, α = 0.781 for coach) was adapted from SDP (2021) and describes the perceived barriers to implementation of the curriculum, with participants describing the challenge of issues such as “Frequent changes in school priorities” and “Teacher turnover.” Unlike with other attribute scales, these items had four response categories (0 = “A great challenge” to 3 = “Not a challenge”).

The curriculum power scale (α = 0.479 for teacher, α = 0.346 for coach) was adapted from SDP (2021) and C-SAIL (2017), and also included several items aligned with policy attribute theory (Porter, 1994) created specifically for R-CAP. This scale addressed the rewards and incentives associated with implementing the curriculum, including items such as “There are negative repercussions for teachers who do not attend professional learning related to the curriculum.” PL was thus indirectly addressed in some of these items—specifically, PL *related to the curriculum*—and so separate PL power items were not collected. This scale is associated with curriculum for the purposes of this study. Because of their low alphas, we note below our caution regarding findings directly related to the teacher and coach curriculum power scales.

#### Aggregate PL and curriculum quality scales and divergence scales

We were also interested in understanding participants’ perceptions of the PL program and curriculum quality overall. To this end we created an aggregate PL attributes scale by taking the mean of the three PL attributes for all participants with available data (*n* = 424 for teachers, *n* = 69 for coaches), and an aggregate curriculum attributes scale by taking the mean of the six curriculum attributes for all participants with data on these scales (*n* = 415 for teachers, *n* = 69 for coaches). In the case of curriculum stability (a 0–3 scale), the scale results were multiplied by $\:\frac{5}{3}$ to weight equally with the other 0–5 scales.

These aggregate attribute scales reflected the participants’ overarching perceptions of the quality of their PL program and curriculum materials and allowed us to also explore the extent to which teachers and coaches shared similar or distinct perceptions. Using the hierarchical dataset, we also created standardized “divergence” scales for both PL and curriculum scales by taking the difference between each teacher’s aggregate scores and that of the coach at their school and standardizing both scores. These scales could then be used to determine whether teacher perceptions about the quality of their coach or coach-reported instructional activities predicted the extent to which teachers and coaches disagreed (or diverged) in their perceptions of the PL program and/or curriculum.

#### Teacher perceptions of coach quality

We also investigated teachers’ perceptions of the quality of their coach. We included items adapted from the New York State Education Department (NYSED, 2014), Castillo et al. (2016), and the New Teacher Center (Young et al., 2017) for these scales, focusing on perceptions of coach expertise (α = 0.866; e.g., “I have confidence in my coach’s knowledge about effective teaching strategies”) and the coach’s affective qualities (α = 0.929; e.g., “My coach is respectful and collegial”).

#### Coaching activities

We used coach-reported scales of curriculum use frequency, coaching emphasis on content and pedagogical knowledge, and coaching emphasis on lesson planning and alignment. Coach reliance on curriculum materials can lead to surface-level teacher engagement with such materials if the focus is primarily on integrating the materials into instruction and planning but can also lead to high-depth teacher engagement if the focus is on *how* the materials support student learning (Coburn & Russell, 2008). Given that PL programs that focus on the introduction of new, high-quality curriculum materials have been found to positively impact student achievement (Lynch et al., 2019), and given the focus on curricula adoption in the R-CAP PL programs, this facet of coaching appeared worthwhile to explore further.

Coburn and Russell’s (2008) caveat about connecting the use of curriculum materials to *how* students learn informed the other coaching activities we chose to investigate: First, we looked at activities focused on developing teachers’ content and pedagogical knowledge. Such activities are also recognized as important mathematics coaching practices (Gibbons & Cobb, 2017; Lynch et al., 2019). Second, we looked at activities emphasizing lesson planning and curriculum. Such activities are of interest because of the explicit focus in these PLPs on implementing specific curriculum materials.

Our coach curriculum materials use scale (α = 0.850) was aligned with Blazar et al. (2020) and included items such as “About how often do you use the curriculum to support teachers in choosing activities for their lessons?” For our scales on coaches’ reported emphasis on content/pedagogical knowledge (α = 0.736) and lesson planning/alignment (α = 0.793) we drew from coaching program evaluations (Clear Creek, 2018). These scales asked coaches to identify their emphasis (“no emphasis” to “a major emphasis”) on different activities that they used with a typical teacher (i.e., one who requires an average amount of support), including items such as “Deepening teacher knowledge of instructional strategies” for the content/pedagogical knowledge scale and “Analyzing and planning lessons” for the planning/alignment scale.

### Analysis

We conducted descriptive and inferential analyses to answer our research questions. We used independent samples t-tests to answer the first and second research questions, and we used OLS regressions and hierarchical linear modeling (HLM) to address the third research question. All analyses were conducted using IBM’s SPSS version 27.

#### RQ1: Alignment of teacher and coach perceptions of PL program and curriculum

For the aggregate dataset, we conducted t-tests to determine whether significant differences existed in the means between coaches and teachers across the PLPs in our study for policy attributes of PL and curriculum. Because the population of teachers was nearly six times the size that of the coaches, equal variance was not assumed for any t-tests. This is because Type I error protection can be optimized by “using a separate-variances test unconditionally whenever sample sizes are unequal” (Zimmerman, 2004, p. 173). Additionally, we applied the Bonferroni-correction when identifying the significance of all *p* values, as this helps avoid the inclusion of false positives from repeated t-tests (Kaltenbach, 2012). Finally, we calculated Hedges’ *g*, an estimator of effect size which is similar to Cohen’s *d* but corrects for biases from small sample sizes (Hedges & Olkin, 1985).

#### RQ2: Perceptions of teachers based on presence of coach

In addition to testing for differences in teacher and coach responses to policy attributes, we were also curious about whether teachers’ interaction with coaches resulted in different perceptions about the quality of the PL program and curriculum. To this end we conducted t-tests that compared teachers who indicated that they worked with a coach (*n* = 121) to those who indicated that they did not (*n* = 294) to help learn about and implement their curriculum. Because sample sizes were not consistent across these groups, we again opted not to assume equal variance for this analysis.

#### RQ3: Coaching aspects that predict teacher perceptions of PL and curriculum quality

We used the hierarchical dataset for our third research question, looking at how teacher-reported perceptions about their coach and coach-reported activities predicted teacher perceptions about the quality of the PL program and curriculum. Because teacher responses about their coach’s expertise and affective qualities were only composed of teacher-level data, we used OLS regression to examine this aspect. Additionally, because the expertise and affective quality agreement scales were found to not be normally distributed, we chose to focus our analysis on teachers with “high” perceptions of coaches. We created a dichotomous variable where the maximum scale scores were given a value of 1. This resulted in 71 teachers (out of 151) with high perceptions of coach expertise and 74 teachers with high perceptions of coach affective quality. It is worth noting that both high perception variables were highly correlated among teachers (*r* =.483, *p* <.001).

Next, we used HLM to look at whether coach-reported emphasis with the relevant coaching activities (planning and alignment, content and pedagogical knowledge, use of the curriculum materials; level 2 data) predicted teacher perceptions about the quality of the PL and curriculum as well as teacher-coach agreement (or divergence) regarding such perceptions (level 1 data). HLM is better able to represent cross-level interactions (Osborne & Neupert, 2013) and account for data which is clustered in some way (Keith, 2019). For each model with level 2 (coach level) data, these predictors were added as fixed effects, and for all models a maximum likelihood estimation model was selected. All variables were grand-mean centered and standardized before analysis for interpretation to emphasize differences across relative teacher and coach responses within the sample. To indicate the presence of clustering, Intraclass Correlation Coefficients (ICCs) were calculated on unconditional models of relevant dependent variables (teacher PL perceptions, teacher-coach PL perceptions divergence, teacher curriculum perceptions, and teacher-coach curriculum perceptions divergence).

## Results

### RQ1: Alignment of teacher and coach perceptions of PL program and curriculum

The descriptive statistics for the teacher and coach attribute scales are shown in Tables 2 and 3, while *t*-tests comparing teacher and coach means for these surveys are shown in Table 4. For both the aggregate scales measuring PL program attributes [*t*(127) = 7.05, *p* <.001] and curriculum attributes [*t*(110) = 7.54, *p* <.001], coaches had significantly higher perceptions that these attributes were in place than teachers, with a Bonferroni adjusted alpha level of 0.005 (0.05/11). When examining each individual attribute, we also found that coaches across the eight PLPs reported significantly higher perceptions than teachers about the presence of every attribute for both the PL program and curriculum.

**Table 2:** Descriptive statistics for scales from R-CAP teacher survey

Scale	*N*	Mean	SD	Min	Max	Skewness
PL Attributes (Aggregate)	424	3.64	0.95	0.33	5.00	−1.17
Specificity	432	3.77	1.13	0.00	5.00	−1.43
Consistency	427	3.42	0.94	0.00	5.00	−0.67
Authority	424	3.73	1.07	0.00	5.00	−1.28
Curriculum Attributes (Aggregate)	415	3.15	0.61	0.68	4.60	−0.53
Specificity	424	3.35	0.80	0.63	4.75	−1.15
Consistency	421	3.99	0.89	0.00	5.00	−1.26
Institutional Authority	417	3.27	0.86	0.00	5.00	−1.00
Normative Authority	417	2.75	0.92	0.25	5.00	0.02
Stability	415	1.91	0.66	0.00	3.00	−0.52
Power	415	2.69	0.83	0.00	5.00	−0.08

All scales range from 0–5 except for Curriculum Stability, which ranges from 0–3

**Table 3:** Descriptive statistics for scales from R-CAP coach survey

Scale	Mean	SD	Min	Max	Skewness	
PL Attributes (Aggregate)	4.26	0.62	2.60	5.00	−0.60	
Specificity	4.25	0.70	2.60	5.00	−0.68	
Consistency	4.17	0.74	2.00	5.00	−0.65	
Authority	4.35	0.68	2.50	5.00	−0.98	
Curriculum Attributes (Aggregate)	3.64	0.47	2.62	4.57	−0.11	
Specificity	4.01	0.66	2.13	5.00	−0.41	
Consistency	4.32	0.58	2.71	5.00	−0.84	
Institutional Authority	3.76	0.66	1.57	4.86	−0.50	
Normative Authority	3.14	0.88	1.29	5.00	0.19	
Stability	2.40	0.41	1.09	3.00	−0.90	
Power	3.00	0.62	1.38	4.38	−0.23	

All scales range from 0–5 except for Curriculum Stability, which ranges from 0–3

Coaches across the eight PLPs perceived greater amounts of PL specificity [*t*(133) = 4.83, *p* <.001], consistency [*t*(106) = 7.54, *p* <.001], and authority [*t*(131) = 6.35, *p* <.001] as well as curriculum specificity [*t*(104) = 7.45, *p* <.001], consistency [*t*(127) = 3.96, *p* <.001], institutional authority [*t*(110) = 5.46, *p* <.001], normative authority [*t*(95) = 3.43, *p* 0.001], stability [*t*(135) = 8.23, *p* <.001], and power [*t*(114) = 3.67, *p* <.001], compared to teachers. This means that coaches perceived both the PL program and the curriculum materials to be of higher quality than what the teacher participants perceived.

**Table 4:** t-Test results of mathematics teacher and coach survey scales

	Mean Diff	t	df	*p*	Hedges’ g
PL Attributes (Aggregate)	0.62	7.05	127	< 0.001	0.91
Specificity	0.48	4.83	133	< 0.001	1.08
Consistency	0.76	7.54	106	< 0.001	0.92
Authority	0.62	6.35	131	< 0.001	1.03
Curriculum Attributes (Aggregate)	0.49	7.54	110	< 0.001	0.60
Specificity	0.66	7.45	104	< 0.001	0.78
Consistency	0.33	3.96	127	< 0.001	0.85
Institutional Authority	0.49	5.46	110	< 0.001	0.84
Normative Authority	0.40	3.43	95	< 0.001	0.92
Stability	0.48	8.23	135	< 0.001	0.63
Power	0.31	3.67	114	< 0.001	0.80

**Fig. 1:**
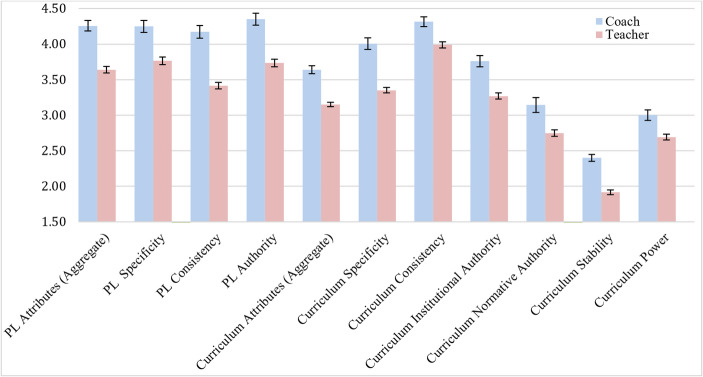
Coach and teacher agreement regarding PL and curriculum attributes. All scales range from 0–5 except for Curriculum Stability, which ranges from 0–3

These results show that coaches perceived greater quality across all attributes regarding both PL and curriculum. However, as shown in Fig. 1, the mean differences between teacher and coach responses were moderate, with a mean difference of 0.62 for the aggregated PL attributes and 0.49 for the curriculum attributes. This indicates that teachers and coaches held significantly different perceptions of PL and curriculum, but not wholly distinct perceptions.

### RQ2: Perceptions of teachers based on presence of instructional coach

Teachers who reported working with an instructional coach also reported significantly different perceptions than those who did not (see Table 5). For both the aggregated PL attributes [*t*(188) = 4.27, *p* <.001] and curriculum attributes [*t*(224) = 3.80, *p* <.001], teachers with instructional coaches reported these to be of higher quality than teachers without a coach. Teachers who reported working with a coach had significantly higher perceptions of the PL program’s specificity [*t*(188) = 3.69, *p* <.001], consistency [*t*(196) = 3.75, *p* <.001], and authority [*t*(191) = 4.20, *p* <.001] as well as the curriculum’s specificity [*t*(189) = 3.44, *p* <.001], consistency [*t*(193) = 3.36, *p* <.001, institutional authority [*t*(200) = 4.09, *p* <.001], and normative authority [*t*(230) = 3.43, *p* <.001]. In sum, teachers who reported having coaching support with the content of the PL and curriculum also felt that both the PL program and the curriculum were better detailed, beneficial to their instruction, and aligned with their school and district priorities. Perceptions of curriculum stability and power were not significantly different between teachers who did and did not report working with a coach. Given the low reliability of the curriculum power scale, this latter finding should be considered with caution.

**Table 5:** t-Test results of teachers who reported working with and without a coach

	With Coach (*N* = 294)		Without Coach (*N* = 121)		Mean Diff				Hedges’ g
	M	SD	M	SD		t	df	*p*	
PL Attributes (Aggregate)	3.77	0.86	3.31	1.07	0.47	4.27	188	< 0.001	0.93
Specificity	3.91	1.04	3.43	1.28	0.48	3.69	188	< 0.001	1.11
Consistency	3.53	0.88	3.13	1.03	0.40	3.75	196	< 0.001	0.93
Authority	3.88	0.99	3.36	1.20	0.52	4.20	191	< 0.001	1.06
Curriculum Attributes (Aggregate)	3.28	0.61	3.03	0.61	0.25	3.80	224	< 0.001	0.61
Specificity	3.44	0.74	3.12	0.91	0.32	3.44	189	0.001	0.79
Consistency	4.09	0.83	3.75	0.98	0.34	3.36	193	0.001	0.88
Institutional Authority	3.38	0.81	2.99	0.92	0.39	4.09	200	< 0.001	0.85
Normative Authority	2.84	0.92	2.51	0.89	0.33	3.43	230	< 0.001	0.91
Stability	1.91	0.67	1.93	0.64	−0.02	−0.32	232	0.746	0.66
Power	2.73	0.83	2.58	0.81	0.15	1.74	229	0.084	0.83

All scales range from 0–5 except for Curriculum Stability, which ranges from 0–3

### RQ3: Coaching aspects that predict teacher perceptions of PL and curriculum quality

### Teacher perceptions of coach quality

OLS regression was used to test if teacher perceptions of their coach’s expertise and affective qualities significantly predicted the teachers’ perception of their PL program’s quality. The regression was statistically significant, (*F*[2, 148] = 13.549, *MSE* = 0.644, *p* <.001), with an R^2^ of 0.155. High ratings of perceived coach expertise (β = 0.272, *p* =.002) and coach affective qualities (β = 0.181, *p* =.038) were both statistically significant predictors of teacher perception of PL quality.

Similarly, we tested if teacher perceptions of their coach’s expertise and affective qualities significantly predicted teachers’ perception of their curriculum’s quality. This regression was also statistically significant, (*F*[2, 148] = 18.356, *MSE* = 0.287, *p* <.001), with an R^2^ of 0.199. High ratings of perceived coach expertise (β = 0.308, *p* <.001) and coach affective qualities (β = 0.207, *p* =.015) were both statistically significant predictors of teacher perception of curriculum quality.

### Coaching activities and PL program perceptions and divergence

Because coach data in our hierarchical dataset were at the school level, we used HLM to understand relationships between coaching practices and teacher aggregate PL attribute beliefs (see Table 6). However, the unconditional model indicated insignificant between-school variation (Wald *Z* = 0.741, *p* =.230) and a low ICC (0.044). While this could have allowed for the use OLS regression for this analysis, we chose to continue with the HLM for several reasons.

First, parallel analyses with teacher-coach PL belief divergence (discussed below) indicated significant within- and between-school variation with the data, indicating the viability of an HLM approach. Additionally, accounting for clustering tends to reduce Type I errors regardless (Heck et al., 2013), supporting the use of HLM despite this limitation. Finally, to ensure that the HLM approach did not produce conflicting results with a simple OLS regression, we performed such a regression replicating our loaded model (Table 6, Model 2) to confirm our results. With all of this in mind, we continued with our HLM, loading our fixed effects of the coach’s reported frequency of curriculum use, content/pedagogical knowledge focus, and planning/alignment focus.

**Table 6:** Estimation of coaching activities on teacher PL quality perceptions

Fixed Effects	Model 1 (unconditional)		Model 2	
	*β (SE)*	*t (p)*	*β (SE)*	*t (p)*
Intercept	− 0.024 (0.081)	− 0.297 (0.770)	− 0.029 (0.073)	− 0.396 (0.695)
Curriculum Use			− 0.204 (0.066)	−3.106 (0.005)
Knowledge Focus			0.007 (0.099)	0.072 (0.943)
Planning and Alignment Focus			− 0.068 (0.105)	− 0.655 (0.517)
Random Effects (variance components)	*Variance*	*Z (p)*	*Variance*	*Z (p)*
Within school variation	0.9522	8.831 (< 0.001)	0.9403	9.021 (< 0.001)
Between school variation	0.0440	0.741 (0.230)	0.0038	0.093 (0.926)

As shown in Table 6, only coach-reported frequency of using the curriculum in their coaching practices predicted (negatively) teachers’ perception of the PL program quality (β = − 0.204, SE = 0.066, *p* =.005). This is in line with the confirmatory regression model run for these three coaching practices on teacher PL attribute beliefs, *F*(3, 187) = 3.35, *MSE* = 0.964, *p* =.020, where coach’s curriculum use was also the only statistically significant predictor (β = − 0.202, SE = 0.065, *p* =.002).

Next, we investigated the relationship between coaching activities and teacher-coach disagreement/divergence concerning the quality of the PL program, as shown in Table 7. The unconditional model for teacher-coach divergence showed significant within school (Wald *Z* = 8.99, *p* <.001) and between school (Wald *Z* = 2.99, *p* =.002) variation, along with a large ICC of 0.324. This indicated the presence of clustering, confirming the applicability of the HLM approach.

When loading the three coaching effects (Model 2), both coach-reported frequency of curriculum use (β = 0.360, SE = 0.076, *p* <.001) and coach focus on content and pedagogical knowledge (β = 0.309, SE = 0.113, *p* =.010) predicted a divergence in perceptions about the PL program quality between the teacher and their coach. Additionally, the ICC of this second model dropped to 0.122, indicating that about 20% of the variance with the unconditional model was accounted for with the added effects.

**Table 7:** Estimation of coaching activities on teacher-coach PL perception divergence

Fixed Effects	Model 1 (unconditional)		Model 2	
	*β (SE)*	*t (p)*	*β (SE)*	*t (p)*
Intercept	0.032 (0.117)	0.273 (0.786)	0.051 (0.082)	0.629 (0.533)
Curriculum Use			0.360 (0.076)	4.711 (< 0.001)
Knowledge Focus			0.309 (0.113)	2.738 (0.010)
Planning and Alignment Focus			− 0.052 (0.115)	− 0.449 (0.656)
Random Effects (variance components)	*Variance*	*Z (p)*	*Variance*	*Z (p)*
Within school variation	0.6522	8.99 (< 0.001)	0.6471	9.05 (< 0.001)
Between school variation	0.3130	2.99 (0.002)	0.0895	1.82 (0.035)

### Coaching activities and curriculum quality perceptions and disagreement

In addition to examining the relationship between coaching activities and teacher PL quality perceptions, we also investigated this relationship with regards to teacher perceptions of the curriculum quality. The unconditional model for teacher aggregate curriculum attribute ratings indicated statistically significant within school variation (Wald *Z* = 8.87, *p* <.001), and between school variation which approached statistical significance (Wald *Z* = 1.60, *p* =.054). For similar reasons as described above with the PL perceptions, and because the ICC for this unconditional model (0.120) indicated a fair amount of clustering, HLM was used to analyze these data. The results of this model (Table 8) show that only coach-reported frequency of curriculum use in their coaching activities predicted (negatively) teachers’ perceptions about the quality of the curriculum (β = − 0.262, SE = 0.075, *p* =.002), a similar finding as the PL quality model.

**Table 8:** Estimation of coaching activities on teacher curriculum perceptions

Fixed Effects	Model 1 (unconditional)		Model 2	
	*β (SE)*	*t (p)*	*β (SE)*	*t (p)*
Intercept	− 0.057 (0.094)	− 0.609 (0.547)	− 0.061 (0.082)	− 0.741 (0.464)
Curriculum Use			− 0.262 (0.075)	−3.498 (0.002)
Knowledge Focus			− 0.044 (0.112)	− 0.393 (0.697)
Planning and Alignment Focus			− 0.076 (0.116)	− 0.657 (0.515)
Random Effects (variance components)	*Variance*	*Z (p)*	*Variance*	*Z (p)*
Within school variation	0.8811	8.87 (< 0.001)	0.8631	9.05 (< 0.001)
Between school variation	0.1207	1.60 (0.055)	0.0529	1.07 (0.142)

Finally, we investigated the relationship between coaching activities and the extent to which teachers and their coach disagreed about the quality of the curriculum, as shown in Table 9. The unconditional model for teacher-coach divergence showed significant within school (Wald *Z* = 8.88, *p* <.001) and between school (Wald *Z* = 2.91, *p* =.002) variation, along with a large ICC of 0.356, indicating the presence of clustering.

When loading the three coaching effects (Model 2), both coach-reported frequency of curriculum use (β = 0.232, SE = 0.087, *p* =.013) and coach focus on content and pedagogical knowledge (β = 0.397, SE = 0.127, *p* =.004) predicted a divergence in perceptions about the quality of the curriculum between the teacher and their coach. Additionally, the ICC of this second model dropped to 0.181, indicating that about 18% of the variance with the unconditional model was accounted for with the added effects.

**Table 9:** Estimation of coaching activities on teacher-coach curriculum perception divergence

Fixed Effects	Model 1 (unconditional)		Model 2	
	*β (SE)*	*t (p)*	*β (SE)*	*t (p)*
Intercept	− 0.023 (0.123)	− 0.187 (0.853)	− 0.005 (0.092)	− 0.056 (0.956)
Curriculum Use			0.232 (0.087)	2.665 (0.013)
Knowledge Focus			0.397 (0.127)	3.128 (0.004)
Planning and Alignment Focus			0.015 (0.128)	0.909 (0.909)
Random Effects (variance components)	*Variance*	*Z (p)*	*Variance*	*Z (p)*
Within school variation	0.6536	8.88 (< 0.001)	0.6471	8.95 (< 0.001)
Between school variation	0.3613	2.91 (0.002)	0.1429	2.16 (0.016)

## Discussion

Despite the increasing ubiquity of coaching as a feature of PL programs (Darling-Hammond, 2017; Kraft et al., 2018), coaches often operate as hidden players in research related to such PL (Hjalmarson & Baker, 2020). Our investigation sheds light on the nature of the teacher and coach perceptions of curriculum-embedded mathematics PL and the associated curricula. This work also indicates that (1) how teachers view the quality of their coach and (2) what activities the coaches focus on impacts teacher perceptions of their PL and curriculum quality. Given that curriculum-embedded PL is a key element of successful STEM PL programs (Lynch et al., 2019), and that this was an explicit priority of PLPs participating in R-CAP, these findings may also be especially beneficial to policy actors employing coaching to support teacher learning and instructional change linked to particular curriculum materials. Because countries with strong foundations of educational professionalism are increasingly promoting job-embedded coaching as a lever of teacher education (Darling-Hammond, 2017), our findings may be relevant to policy actors in those jurisdictions and to actors in places such as the United States hoping to develop similar educational systems.

Our first research question sought to understand the differences in how mathematics teachers and coaches perceive of their district’s PL program and curriculum quality. Coaches in our sample perceived the PL programs and curriculum to be of higher quality than teachers did for each policy attribute: specificity, consistency, and authority (for PL), and specificity, consistency, institutional authority, normative authority, stability, and power (for curriculum). Some of these attributes (e.g., normative authority or buy-in) may have been more prone to specific role experiences and subjective beliefs about the PL and curriculum. However, even for attributes such as specificity of the curriculum, which presumably consists of the same materials, coaches perceived the program and materials to be of higher quality than teachers did within these PLPs.

This indicates that teachers and coaches held distinct perceptions about structures (e.g., PL and curricula) that informed their shared work. This could be because coaches were more bought-in to the sort of ambitious instruction that is intended to drive such learning and curriculum implementation (e.g., Campbell & Malkus, 2014; Kane & Saclarides, 2022). It could also be that teachers and coaches were engaging with the PL activities and curricula in distinct communities of practice, with teacher-coach interactions limited by policy level factors such as school leader participation and buy-in to the work (Coburn & Russell, 2008; Kane et al., 2018; Shirrell et al., 2019). Regardless, given that coaches support teachers’ instructional change in part by lending their expertise to moderate teachers’ interactions with one another (Coburn & Russell, 2008; Hopkins & Woulfin, 2015; Sun et al., 2014), this disconnect warrants consideration in the development of educational infrastructures such as the PLPs described in this study. These results could underscore the importance of ensuring opportunities for coaches to foster “pedagogical empathy” (Kane & Saclarides, 2023, p. 9) with the teachers whom they support, or to at least seek out and better understand their teachers’ instructional and learning perspectives and how those might influence their shared work.

Our second research question explored how teacher views varied based on whether or not they worked with a coach. Teachers who were supported by coaches in implementing the curriculum and learning of the PL program described both the PL and curriculum to be of higher quality than those who were not coached, across attributes of specificity, consistency, and authority. Indeed, the only attributes where these two groups of teachers did not have significantly different results were with regard to the curriculum’s stability and power.

This indicated that something about the coaching role—or the PLPs that chose to use this lever of PL—may have supported teachers’ experience with the PL and the curriculum and brought them more toward agreement with their coaches’ higher perceptions of quality for these components. Just as Coburn and Russell (2008) found that the depth of interaction that coaches had with the content and curriculum of their PL impacted teachers’ engagement with their own learning, these results could have suggested that the coach role can offer an additional lever of support in fostering more positive teacher engagement with the PL and curriculum. Although our overall findings indicated that coaches perceived the PL and curriculum to be of higher quality, these particular findings reaffirmed a positive, reciprocal relationship between PL and coaching with regards to teacher perceptions of their instruction and learning.

In these ways, our first two research questions offered mixed results: Teachers had a lower opinion about the quality of their PL and curriculum than did coaches, but the presence of a coach in supporting teachers’ engagement with their curriculum-embedded PL at least lessened that divide. Our third research question helped us to better make sense of these findings, with results suggesting possibilities for how teachers’ perceptions of their coach’s quality, and coaches’ choice of instructional activities, can both play a role in the coaching experience.

First, teachers who reported that their school-based coaches held the highest levels of expertise in mathematics content and pedagogy—as well as affective qualities such as creating an environment of trust—also believed more strongly in the quality of their PL and curriculum. One potential reading of our results is that teachers with strong beliefs about their coach’s quality may also be more open to the structures and goals of curriculum-embedded PL programs.

These teachers may also have had coaches who truly did exhibit high amounts of content expertise and affective qualities, which may have helped the coaches to facilitate productive mathematics coaching activities (e.g., Gibbons & Cobb, 2017) and attend to teacher resistance (e.g., Kane & Saclarides, 2022; Russell et al., 2020). If these school-based coaches acted as a “face” of the PL program for their teachers, those teachers’ perceptions of the PL program and of their coach might also have been entangled. Thus, a positive perception of a coach’s quality could have also cast a positive light on the program that the coach was supporting. Such quality coaches might also have enabled teachers to experience more of the indicators of high-quality PL, just as Coburn and Russell (2008) found that teachers tended to replicate the routines of interactions they had with their coaches in their own social networks.

Interestingly, teachers with less positive perceptions about the quality of their curriculum and PL—and with greater divergence from the perceptions held by their coaches—worked with coaches who reported using the curriculum materials at greater frequencies. Although this may seem counterintuitive, there are several possible explanations for why this may be the case. One explanation is that, in line with Coburn and Russell (2008), these coaches were being pushed to ensure accountability (e.g., emphasizing pacing, consistency of materials, etc.) rather than to individualize and deepen teachers’ experience with the learning of the PL. If such coaches were using the curriculum materials more frequently but not strategically (e.g., tied to a focus on content and pedagogical knowledge development), their teachers may not have gained more than a transactional relationship with the materials. Ultimately, successfully executing productive mathematics coaching activities requires coach expertise in not only the content but also in facilitating teacher learning (Gibbons & Cobb, 2017). As such, it could have been that these coaches were genuinely implementing the district PL learning and curriculum into their work, but had not yet successfully illustrated the benefit of those policies to their teachers.

Alternatively, these teachers may have had lower opinions of the curriculum and associated PL precisely because their coach had adeptly built this into their work together: the teachers were acutely aware of the limitations of their curriculum and associated PL. Thus, teachers may have had less positive perceptions of the curriculum and the curriculum-embedded PL not because they had a surface level understanding of these components, but precisely because they had a deeper understanding of it—flaws included.

We also found that while a coach’s focus on strengthening content and pedagogical knowledge did not predict teachers’ perceptions of the PL and curriculum quality, it did predict a *divergence* of such perceptions between the teacher and their coach. This result could indicate that coaches relied more on building such knowledge when they recognized a mismatch between their own experiences with the curriculum and PL and those of their teachers. In other words, focusing explicitly on content and pedagogical knowledge—in addition to curriculum use—may have been seen by coaches as a way to build understanding and buy-in with their teachers. This would contrast with Kane and Saclarides (2022), where coaches often described teacher resistance as a *barrier* to their willingness to enact certain coaching activities. Given that the focus on such knowledge and the divergence in teacher-coach perceptions of the PL program and curriculum quality was occurring at the same time in our data, it remains to be seen whether this observed approach might lessen that gap over time.

Additionally, because these data were from a single point in time, it may not be the case that coaches were responding to a perceived gap at all—the gap may have been arising concurrently with the coach’s focus on content and pedagogical knowledge or may have been arising in *response* to this coaching focus. Either possibility is notable, as they both cast doubt on the effectiveness of using coaching to strengthen the implementation of curriculum-embedded PL programs by focusing on improving teachers’ content and pedagogical knowledge. However, such a reading could also support the coaching model proposed by Kraft et al. (2018), which suggests that teachers best develop content and pedagogical knowledge in group PL prior to engaging in individualized coaching activities. In either interpretation, these findings strengthen the idea that the focus of coaching and the timing of that coaching are both important considerations in designing effective mathematics PL programs that incorporate coaching. Given many countries’ evolving educational policies aimed integrating coaching into ongoing, school-embedded teacher development (Darling-Hammond, 2017), these results indicate that such policies should pay close attention to both *when* and *how* coaching is enacted.

Our findings should also be interpreted through both our study’s strengths and limits. While our sample of teachers and coaches reflected experiences of teachers in diverse urban districts across the United States, the sample is not representative beyond that scope. Further, the participants were in districts that were actively engaging in curriculum-embedded PL, and any interpretation of the data should keep these constraints in mind. Additionally, our findings were based on survey data. While our questions are behavioral-based, increasing their validity and reliability (Mayer, 1999), observations and artifacts of teacher-coach interactions would deepen our ability to examine the nature and quality of interactions.

A broader complication of our data is that each PLP had varying numbers of teachers and coaches. Because the sample size of our data was insufficient for directly comparing policy attributes for teachers and coaches for each PLP and curriculum, our methodology leaves open the possibility that some other factors besides coaching may have been driving the results. Our study was also cross-sectional; future work would benefit from examining how coach-teacher relationships evolve over time.

Finally, it is notable that this survey was conducted in the Winter of 2020–2021. Although this time point is not at the onset of the worldwide COVID-19 pandemic, ongoing educational issues related to the pandemic could have driven teacher and coach responses to this survey. However, through other data collected for the R-CAP project, we documented that the shift to online PL and coaching, while a technological challenge initially, allowed PLPs to provide the level and intensity of PL that they had planned, with similar activities and engagement, and in fact, several PLPs reported that interactions were easier to schedule virtually than in person (Hill et al., 2025). Indeed, it is noteworthy that—despite the circumstantial challenges of the COVID-19 pandemic—the mean aggregated perceptions for both coaches and teachers about the quality of the PL (mean of 4.26 and 3.64, respectively) and curriculum (mean of 3.64 and 3.15, respectively) fell above the “somewhat agree” Likert scale score of 3. Simply put, these data indicate that participants broadly found the PL and curriculum to be quality elements of the PLPs. Additionally, Baker et al. (2022) found that mathematics specialists’ primary responsibilities tended to be supporting teachers’ PL and curriculum implementation both before and during the pandemic, and that such coaches strengthened pandemic-related instructional shifts (such as moving to online learning). Thus, we also conjecture that these data were collected at a time when the importance of teacher coaching support was actually heightened.

## Conclusion

In our analysis of middle school mathematics teachers and coaches engaged in curriculum-based instructional reform, we found that coaches, compared to teachers, had significantly more positive views about the quality of these PL programs and curricula. This is consistent with other research showing that leaders tend to have a more positive view of the policy environment than “street-level bureaucrats” (Lipsky, 2010). Most likely, this is at least in part due to teachers facing everyday challenges in implementing the curriculum in their classroom. Understanding and addressing this divergence in perceptions holds promise for designing PL experiences that respect and incorporate the insights and dispositions of teacher participants.

On the positive side, our findings also suggest that working with a school-based coach to effectively implement new curriculum materials—especially a coach whom teachers view as expert, supportive, and trustworthy—improves teachers’ views about the quality of their own learning and the curriculum they are using. Thus, our findings here provide promising evidence of a coach’s role in building mathematics teachers’ buy-in (authority) for their PL and curriculum, understanding of the PL activities and curriculum content (specificity), and recognition of the relationship of the PL and curriculum with other programs and policies (consistency).

At the same time, our findings also reflect the complexity of the relationship between a mathematics coach’s chosen activities and their teachers’ perceived experience. Even when coaches focus their work explicitly on using the curriculum and developing their teachers’ content and pedagogical knowledge, this does not—at least immediately—bring teachers on board with the coach’s perceptions of the district-mandated PL and curriculum. Thus, we conclude that including coaching as a lever for teacher change in mathematics PL programs should be considered with care. Coaching can be a powerful tool for supporting ongoing professional learning but should not be assumed as a magic bullet for achieving programmatic goals. We concur with Hjalmarson and Baker (2020) that coaches operate as valued members of teachers’ learning communities and warrant explicit consideration as participants in and facilitators of PL programmatic learning goals. Our findings indicate the value that coaches can add to such PL programs while also suggesting that close attention must also be paid to the timing and nature of such coaching. If district PL and curriculum initiatives aim to be seen as high-quality by teacher and coach participants, then the perspectives of these participants deserve to be recognized, responded to, and respected.
